# Association between physical activity and the prevalence of gout among patients with type 2 diabetes mellitus and hyperuricemia: a two-center population-based cross-sectional study

**DOI:** 10.1007/s10067-024-07081-5

**Published:** 2024-07-26

**Authors:** Ningyu Cai, Mengdie Chen, Lijing Wu, Ping Feng, Xun Ye, Qiao Liu, Xianping Zhu, Chaoyin Lu, Qidong Zheng, Yiyun Wang

**Affiliations:** 1 Department of Endocrinology, Taizhou Central Hospital (Taizhou University Hospital), Taizhou, 318000 Zhejiang China; 2 Department of Orthopedics, Taizhou Central Hospital (Taizhou University Hospital), Taizhou, 318000 Zhejiang China; 3 Department of Internal Medicine, Yuhuan Second People’s Hospital, No.77, Environmental Protection Middle Road, Chu Men Town, Yuhuan, 317600 Zhejiang China; 4 Department of Endocrinology, Hangzhou Hospital of Traditional Chinese Medicine, Hangzhou, 310007 Zhejiang China

**Keywords:** Gout, Hyperuricemia, Physical activity, Type 2 diabetes mellitus

## Abstract

**Introduction:**

Diabetes mellitus (DM) and gout cohabitation severely reduces patient life quality while raising financial burden on individual and society. The aim of this study was to elucidate the association between physical activity (PA) and the prevalence of gout among type 2 DM (T2DM) and hyperuricemia (HUA) patients.

**Methods:**

In all, we recruited 2291 T2DM patients with HUA. Among them, 448 had gout and 1843 did not. We collected patient data, such as anthropometry, laboratory reports, and medical history, for our analyses. The PA assessment was based on the Chinese version of International PA Questionnaire-short (IPAQ). Moreover, the relationship between PA and gout risk was examined using multivariate logistic regression models.

**Results:**

Total PA was markedly low among gout patients, relative to controls (*p* < 0.05). Based on the IPAQ categorical score, 38.2% exhibited “low,” 26.8% “moderate,” and 35.0% “high” PA among gout patients. In comparison, 12.4% performed “low,” 53.8% “moderate,” and 33.8% “high” PA among controls. Multivariate analysis revealed that, after adjustment of confounding factors, both low (OR 6.382) and high PA (OR 2.048) had a higher prevalence of gout, as compared to moderate PA. Moreover, we revealed that the male sex, age, HUA duration, serum uric acid, glycated hemoglobin, dyslipidemia history, and drinking status were also independent indicators of the prevalence of gout. Furthermore, stratification analyses revealed results consistent with our prior results.

**Conclusions:**

PA intensity was associated with the prevalence of gout among T2DM and HUA patients, and the lowest prevalence was achieved from moderate intensity PA.

**Table:** 

**Key Points**
• *PA intensity was associated with the prevalence of gout among T2DM and HUA patients*.
• *The lowest prevalence of gout was achieved from moderate intensity PA*.

## Introduction

Gout is relatively common among patients suffering from inflammatory arthritis, and it is the result of monosodium urate (MSU) crystal deposition within joints and tissues, once the circulating MSU levels rise beyond the saturation point of the crystal [1]. More recently, gout frequencies have increased in several locations and nations [2]. Globally, gout incidences rose from 0.08 to 2–4% between 2010and 2020 [3]. One investigation revealed that the weighted gout incidence rate was 3.2% (4.4% for men and 2.0% for women), which amounted to a predicted 25.56 million individuals with gout between 2015 and 2017 among the Chinese adult population [4].

Gout and diabetes mellitus (DM) are often co-occurring conditions [5]. Both DM and gout are intricately linked to enhanced cardiovascular disease (CVD), renal failure, and mortality risks [6]. Moreover, the simultaneous development of gout and DM can severely impact patient quality of life and increase individual and societal financial burden. Hence, it is crucial to examine the delicate association between gout incidence in DM patients and related risk factors. Prior investigations reported that the male sex, advancing age, obesity, dietary factors, hypertension, hyperuricemia (HUA), and genetic susceptibility are potential hazardous factors for gout [1]. Although cumulative studies have been conducted, certain other HUA-related risk factors have not been elucidated.

Physical activity (PA) is described as any physiological action that requires energy expenditure through the skeletal muscle [7]. Augmented PA is strongly correlated with reduced blood pressure (BP), body mass index (BMI), and improvement in glucose metabolism [8].

Given these evidences, PA is a potential underlying factor that influences gout prevalence among DM and HUA patients. However, there are no available reports on this potential regulation. Multiple investigations reported a marked reduction in gout risk with increasing PA [9–12]. However, a bidirectional Mendelian randomization (MR) study indicated that PA potentially reduces circulating MSU contents, but not gout incidence [3].

Herein, the possible associations between PA and the prevalence of gout among T2DM and HUA patients were investigated.

## Materials and methods

### Participants

DM and HUA patients, ≥ 18 years of age, who sought treatment at the National Metabolic Management Center (MMC) [13] in The Second People’s Hospital of Yuhuan and Taizhou Central Hospital (Taizhou University Hospital) between June 2020 and September 2022 were recruited for this investigation. T2DM diagnosis was based on the guidelines of the World Health Organization (WHO) [14]. HUA was identified when serum uric acid (SUA) content was > 420 μmol/L among males or > 360 μmol/L among females [15]. Gout status was determined by a self-report of a prior diagnosis. Type 1 DM and other forms of DM, apart from T2 DM (as described above), were eliminated from the study. Additionally, individuals with recent acute DM complications, such as, ketoacidosis, hyperosmolar hyperglycemic state (HHS), hyperosmolar non-ketotic diabetic coma, and lactic acidosis were also excluded from analyses. After that, a total of 2291 T2DM patients with HUA were enrolled for the study. Subsequently, 448 cases who were diagnosed with gout according to the 1977 American College of Rheumatology preliminary criteria for gout [16] were selected as study subjects, and 1843 without gout were chosen as controls. The study was approved by the local ethic committee, and received written informed consent from all subjects.

### Data collection and PA assessment

We recorded the social demographic, medical history, and lifestyle profiles of all subjects. Patient height and weight were determined using a height-weight scale, while the patients wore light clothing and no shoes. Moreover, BMI was computed as follows: weight/height (kg/m^2^). Each participant's blood pressure(BP) was measured by an automated electronic device. Additionally, blood samples were collected following an overnight (ON) fasting for the measurement of bioindicators, such as fasting blood glucose (FBG), fasting blood C-peptide (FCp), glycated hemoglobin (HbA1c), total cholesterol (TC), triglycerides (TG), high-density lipoprotein cholesterol (HDL-C), low-density lipoprotein cholesterol (LDL-C), serum creatinine (Scr), and SUA. Homeostasis model assessment of insulin resistance (HOMA-IR) was computed as follows: 1.5 + (FCP [pmol/L] × FBG [mmol/L])/2800 [17]. Lastly, the predicted glomerular filtration rate (eGFR) was computed using the equation of the Chronic Kidney Disease Epidemiology Collaboration [18].

The Chinese adaptation of the International Physical Activity Questionnaire-Short (IPAQ) was employed for PA intensity evaluation [19]. The total metabolic equivalent (MET) assessed the energy requirements, and it was computed as follows: MET according to activity type × minutes/day or week. All subjects were asked about the three categories of PA (i.e., walking, moderate activity, and vigorous activity) at the completion of each week. The MET value was calculated by adding up the abovementioned three different ways of PA of the whole week with their corresponding coefficients. The continuous PA indicator is presented as MET-minutes/week. The coefficients of the compiled mean MET were 8 for vigorous activity, 4 for moderate activity, and 3.3 for walking.

PA intensity was stratified into three levels: (1) high-intensity PA (HI-PA), for those individuals whose total PA (including walking and moderate or intense activities) for 7 days per week and reached 3000 MET-minutes/week, or individuals who undertook intense activities for at least 3 days a week and achieved a total PA level of 1500 MET-minutes/week; (2) moderate-intensity PA (MI-PA), for those who engaged in intense activities 20 min per day for a minimum of 3 days per week, or undertook moderate activities or walking for 30 min per day for a minimum of 5 days per week, or who walked and engaged in moderate or intense activities for > 5 days per week and with a total PA level reaching 600 MET-minutes/week; and (3) low-intensity PA (LI-PA), for a person whose some activities were reported, but did not meet the HI-PA or MI-PA grouping criteria described above, or no activity was reported.

### Definitions

Hypertension was described as systolic blood pressure (SBP) ≥ 140 mmHg, diastolic blood pressure (DBP) ≥ 90 mmHg, or a prior hypertension diagnosis [20]. Dyslipidemia was considered when ≤ 1 conditions were met: TC ≥ 5.7 mmol/L, TG ≥ 1.7 mmol/L, LDL-C ≥ 3.6 mmol/L, and HDL-C < 1.29 mmol/L among females, and < 1.03 mmol/L among males [21]. Participants were considered to be smokers and drinkers if they smoked or consumed alcohol daily or nearly daily.

### Statistical analysis

Normally distributed continuous data are provided as means and standard deviation, skewed data as median (interquartile range, IQR), and categorical data as frequency (%). Patient demographics and clinicopathological profiles were evaluated using chi-square test (categorical data) and two-sample *t*-test or Mann–Whitney *U* test (continuous data). We also employed multivariate logistic regression analyses to predict relationship between PA ang the prevalence of gout, using MI-PA as reference. We employed three models for possible confounding factor adjustment. Model 1 received patient age and gender adjustment; model 2 received adjustment for model 1 variables, as well as SUA, HbA1c, and HUA duration; model 3 received adjustment for all model 2 variables, as well as Scr, BMI, and lifestyle factors (both smoking and drinking history), along with hypertension and dyslipidemia histories. Additionally, we also conducted stratified analysis on the potential relationship between PA and the prevalence of gout among gender and drinking status subcohorts, using multivariate analyses with complete model 3 adjustment, unless stratified. SPSS 23.0 (IBM) was employed for all data analyses. Two-sided *p*-value < 0.05 was considered statistically significant.

## Results

### The demographic and clinical characteristics of participants

Table 1 summarizes the 2291 participant demographics and clinicopathological profiles. The median (IQR) participant age was 47 (56–63) years, and 1446 were males (63.1%). Moreover, 448 (21.98%), out of 2291 patients, suffered from gout. Relative to subjects without gout, those with gout exhibited prolonged HUA, enhanced FBG, HbA1c, Scr, SUA, TG, FCP, HOMA-IR, BMI, reduced eGFR, and HDL-C, as well as total METs (all *p* < 0.001, Table 1). Moreover, gout sufferers were more often smoking and drinking males, with hypertension and dyslipidemia histories (all *p* < 0.001, Table 1). Lastly, we observed no intergroup differences in patient age, diabetes duration, DBP, SBP, TC, and LDL-C between the two cohorts (Table 1).

**Table 1:** Demographic and clinical parameters of study patients

Variables	Total(*n* = 2291)	With gout(*n* = 448)	Without gout(*n* = 1843)	*p* value
Male, *n* (%)	1446 (63.1)	370 (82.6)	1076 (58.4)	< 0.001
Age (y)	56.00 (47.00–64.00)	56.00 (49.00–65.00)	56.00 (47.00–64.00)	0.286
Duration of HUA (y)	4.38 (2.12–7.46)	4.37 (2.19–9.15)	4.39 (2.11–7.23)	0.02
Duration of diabetes (y)	4.67 (1.00–10.83)	5.08 (1.17–11.08)	4.67 (0.92–10.75)	0.375
SBP (mmHg)	133.00 (123.00–145.00)	134.00 (124.00–147.75)	133.00 (122.00–144.00)	0.133
DBP (mmHg)	76.00 (70.00–85.00)	77.00 (71.00–85.00)	76.00 (69.00–85.00)	0.11
BMI (kg/m^2^)	26.10 (24.00–28.60)	26.35 (24.30–28.70)	26.10 (23.90–28.50)	0.031
Hypertension history, *n* (%)	1545 (67.4)	320 (71.4)	1225 (66.5)	0.044
Dyslipidemia history, *n* (%)	1733 (75.6)	379 (84.6)	1354 (73.5)	< 0.001
Smoking, *n* (%)	551(24.1)	156(34.8)	395(21.4)	< 0.001
Drinking, *n* (%)	477(20.8)	193(43.1)	284(15.4)	< 0.001
FBG (mmol/L)	7.65 (6.36–10.05)	8.11 (6.57–10.77)	7.52 (6.32–9.76)	< 0.001
FCp (ng/mL)	2.50 (1.80–3.37)	2.69 (1.81–3.60)	2.50 (1.80–3.29)	0.017
HOMA-IR	3.84 (3.12–4.95)	4.02 (3.24–5.36)	3.78 (3.10–4.91)	< 0.001
HbA1c (%)	7.50 (6.60–9.30)	7.80 (6.70–9.70)	7.50 (6.60–9.20)	0.011
Scr	72.00 (59.00–89.00)	82.00 (67.00–103.75)	70.00 (57.00–85.00)	< 0.001
e-GFR (µmol/L)	90.73 (70.54–110.70)	82.57 (59.24–105.31)	92.42 (72.75–111.56)	< 0.001
SUA (µmol/L)	437.00 (388.00–483.00)	442.00 (386.00–526.75)	437.00 (388.00–477.00)	0.009
TG (mmol/L)	1.80 (1.23–2.72)	1.94 (1.32–3.10)	1.77 (1.22–2.68)	0.01
TC (mmol/L)	5.15 (4.34–5.97)	5.09 (4.34–5.97)	5.16 (4.33–5.98)	0.883
HDL-C (mmol/L)	1.06 (0.91–1.24)	1.02 (0.85–1.17)	1.07 (0.92–1.26)	< 0.001
LDL-C (mmol/L)	2.87 (2.23–3.56)	2.86 (2.33–3.52)	2.88 (2.20–3.57)	0.514
Physical activity				< 0.001
Total (MET-mins/week)	2772.00 (1247.40–4158.00)	1386.00 (594.00–4158.00)	2772.00 (1386.00–4158.00)	0.009
Low, *n* (%)	400 (17.5)	171 (38.2)	229 (12.4)	
Moderate, *n* (%)	1111 (48.5)	120 (26.8)	991 (53.8)	
High, *n* (%)	780 (34.0)	157 (35.0)	623 (33.8)	

Data presented as median (interquartile range) or *n* (%). Cohort comparisons employed the chi-square and Mann–Whitney *U* tests for categorical and continuous variables, respectively

*HUA*, hyperuricemia; *SBP*, systolic blood pressure; *DBP*, diastolic blood pressure; *BMI*, body mass index; *FBG*, fasting blood glucose; *FCp*, fasting blood C-peptide; *HOMA-IR*, homeostasis model assessment for insulin resistance; *HbA1c*, glycated hemoglobin;* TG*, triglycerides;* TC*, total cholesterol; *HDL-C*, high-density lipoprotein cholesterol; *LDL-C*, low-density lipoprotein cholesterol; *Scr*, serum creatinine; *SUA*, serum uric acid; *eGFR*, estimated glomerular filtration rate; *MET*, metabolic equivalent

Based on the IPAQ score, 38.2% performed LI-PA, 26.8% performed MI-PA, and 35.0% performed HI-PA among gout sufferers. In contrast, among non-gout sufferers, 12.4% performed LI-PA, 53.8% performed MI-PA, and 33.8% performed HI-PA.

### Influencing factors for gout in diabetic patients with HUA

Based on our results, independent gout factors were related to PA (LI-PA: odds ratio (OR) 6.382 (4.738–8.597); HI-PA: OR 2.048 (1.556–2.694); both *p* < 0.001), the male sex (OR 2.295 (1.67–3.154); *p* < 0.001), patient age (OR 1.027 (1.016–1.038); *p* < 0.001), HUA duration (OR 1.036 (1.009–1.064); *p* = 0.008), UA (OR 1.002 (1.001–1.004); *p* = 0.003), HbA1c (OR 1.063 (1.006–1.123); *p* = 0.029), dyslipidemia history (OR 1.754 (1.29–2.385); *p* < 0.001), and drinking status (OR 3.996 (2.972–5.371); *p* < 0.001) (Table 2, model 3).

**Table 2:** Association of the physical activity and other factors with gout in patients with type 2 diabetes mellitus and hyperuricemia

	Model 1		Model 2		Model 3	
	OR (95% CI)	*p* value	OR (95% CI)	*p* value	OR (95% CI)	*p* value
Low	6.264 (4.72–8.313)	< 0.001	5.945 (4.466–7.914)	< 0.001	6.382 (4.738–8.597)	< 0.001
Moderate	Reference		Reference		Reference	
High	2.053 (1.578–2.672)	< 0.001	2.057 (1.578–2.682)	< 0.001	2.048 (1.556–2.694)	< 0.001
Gender (male)	3.913 (2.952–5.188)	< 0.001	3.532 (2.652–4.705)	< 0.001	2.295 (1.67–3.154)	< 0.001
Age	1.021 (1.011–1.031)	< 0.001	1.022 (1.012–1.032)	< 0.001	1.027 (1.016–1.038)	< 0.001
Duration of HUA			1.044 (1.018–1.07)	0.001	1.036 (1.009–1.064)	0.008
SUA			1.002 (1.001–1.004)	0.001	1.002 (1.001–1.004)	0.003
HbA1c			1.057 (1.004–1.114)	0.036	1.063 (1.006–1.123)	0.029
BMI					1.03 (0.997–1.065)	0.079
Hypertension history					1.122 (0.856–1.471)	0.404
Dyslipidemia history					1.754 (1.29–2.385)	< 0.001
Smoking					0.781 (0.58–1.052)	0.104
Drinking					3.996 (2.972–5.371)	< 0.001
Scr					1.001 (1–1.003)	0.075

Model 1 received patient age and gender adjustment; model 2 received adjustment for model 1 variables, as well as SUA, HbA1c, and HUA duration; model 3 received adjustment for all model 2 variables, along with Scr, BMI, lifestyle factors (smoking and drinking histories), as well as hypertension and dyslipidemia histories

*OR*, odds ratio; *CI*, confidence interval; *BMI*, body mass index; *HbA1c*, glycated hemoglobin; *Scr*, serum creatinine; *HUA*, hyperuricemia; *SUA*, serum uric acid; *eGFR*, estimated glomerular filtration rate

### Effects of PA on the prevalence of gout

Table 2 summarizes the relationships between PA intensities and the prevalence of gout, based on the three models. Relative to MI-PA, LI-PA (OR 6.264, 95%CI 4.72–8.313) and HI-PA (OR 2.053, 95%CI 1.578–2.672) were associated with a higher prevalence of gout, following adjustment of patient gender and age (model 1, all *p* < 0.001). Additional adjustment of HUA duration, SUA, and HbA1c (model 2) revealed that the corresponding ORs in the LI-PA and HI-A cohorts were 5.945 and 2.057, respectively (all *p* < 0.001). We conducted further adjustments, including with BMI, hypertension and dyslipidemia histories, and Scr, as well as smoking and drinking statuses, and revealed that the LI-PA and HI-PA remained significant associated with a higher prevalence of gout (model 3, OR of HUA 5.56 and 1.606 for LI-PA and HI-PA, respectively, all *p* < 0.001). For additional details, please refer to Table 2.

**Fig. 1:**
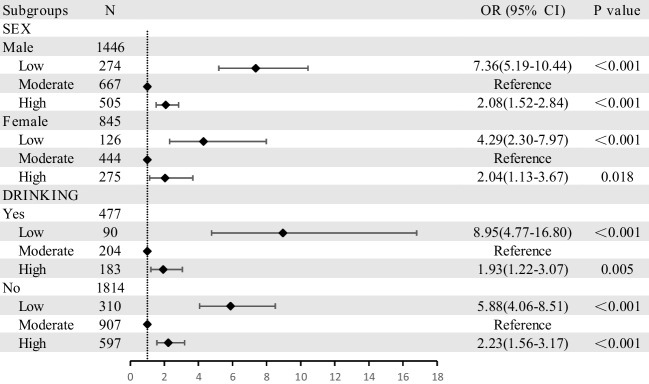
Physical activity and the prevalence of gout subgroup analysis. Adjusted for patient age, gender, SUA, HbA1c, HUA duration, Scr, BMI, and lifestyle factors (smoking and drinking statuses), as well as hypertension and dyslipidemia histories, unless stratified. Abbreviations: OR, odds ratio; CI, confidence interval

Furthermore, since the gender and drinking status were not uniformly distributed among gout and non-gout patients, we next conducted stratified analysis. We completely adjusted for all listed variables in model 3, unless stratified. We revealed results consistent with our prior results (Fig. 1). Moreover, we observed significantly higher corresponding ORs from LI-PA among drinking male patients (8.954 and 7.362, respectively, Fig. 1).

## Discussion

The present study examined the possible relationship between PA and gout among T2DM patients who also suffer from HUA. Our observation revealed that the total PA was significantly decreased among gout patients, relative to patients without gout. Furthermore, compared with MI-PA patients, those with LI-PA or HI-PA were significantly associated with a higher prevalence of gout. Moreover, the relationship remained even after adjustment of potential confounding factors, such as the male sex and drinking status. Given these evidences, the PA intensity strongly modulated gout among T2DM and HUA patients. In contrast, MI-PA produced milder influence on the prevalence of gout, compared to other levels.

However, it is yet unclear how PA impacts gout development and progression. Prior investigations reported that MSU-triggered inflammation induces gout formation, whereas PA promotes anti-inflammatory activities that diminish IL-6, C-reactive protein, and TNF [12, 22, 23]. Additionally, PA strongly eliminated inflammation and gout risk by downregulating macrophage and neutrophil invasion, as well as CXCL1 and TLR2 expressions [10, 11]. Others further reported a series of associated signaling pathways between PA and circulating MSU contents [3]. Studies revealed that MI-PA raises adenosine content and aromatic L-amino acid decarboxylase activity, while activating the renal dopamine system to develop renal diuresis, thereby enhancing urination [24]. It also augments diuretic impact by enhancing the serum taurine content of MSU transporters within the renal proximal tubules [25]. Both aforementioned mechanisms are capable of augmenting MSU excretion.

Owing to diminished excretion and an upregulation of endogenous purine, as previously mentioned, it was possible that the circulating uric acid rose following HI-PA. However, our study observed that relative to LI-PA and HI-PA, MI-PA presented the lowest proportion (26.8%) among gout patients, and the highest proportion (53.8%) among non-gout patients. Furthermore, engaged MI-PA participants exhibited the lowest gout prevalence. Therefore, PA did not appear to be linearly associated with gout incidence among T2DM patients. Given these discrepancies, it is necessary to conduct additional investigations to explore the U-shaped association between various PA intensities and the prevalence of gout.

Multiple prior epidemiological studies reported a strong association between PA and circulating MSU and gout risk. Distinctive observational investigations also indicated that daily PA is highly correlated with diminished circulating MSU content, and inversely related to gout risk [26, 27]. In an 8-week aerobic exercise program, it was confirmed that slow cardiac exercise drastically diminished circulating urate content [27]. A randomized controlled research revealed that LI-PA, as well as prolonged PA, is a potent nonpharmacologic therapy for circulating MSU reduction among hypertensive individuals [28]. A 28.990-person cohort research demonstrated that the gout risk diminished with elevated PA [9]. Additionally, based on a meta-analysis involving 11 trials, the rise in circulating MSU was diminished by 12% among LI-PA and MI-PA, and by 29% among HI-PA [29]. A two-sample MR study examined the causal relationship between PA and circulating MSU levels and gout risk, and revealed that PA significantly reduces circulating MSU content, without affecting gout risk [3].

This investigation has the following strengths. Firstly, we were the first to examine a potential relationship between PA and the prevalence of gout among T2DM and HUA patients. With enhanced gout prevalence, it is imperative to conduct extensive analyses of diabetic patient subsets like HUA, in order to develop targeted therapy, such as distinct PA intensity for delaying gout occurrence and progression. Secondly, we examined a relatively large patient population. Thirdly, herein, we minimized the influence of diabetes, as well as HUA and other influencing factors’ duration, using statistical adjustments.

This research also encountered certain limitations. Firstly, due to its retrospective nature, we could only demonstrate a potential association between PA and the prevalence of gout; however, we were unable to infer causality. We also cannot eliminate the presence of reverse causality (e.g., gout causing joint pain, which leads to restricted PA). Hence, it is essential to conduct prospective investigations among diabetic and hyperuricemia patients, who do not have gout to establish causality in subsequent follow-ups. Secondly, although our study minimized the influence of duration and other factors using statistical adjustments, some confounding factors may still exist, for example, prior treatment history of patient. Thirdly, those included in this investigation were recruited from only two local hospital, and it included only Chinese individuals. Therefore, there may be certain unintentional selection bias. Our results require further validation in a large sample population. Moreover, the PA evaluation was self-reported; hence, there may be certain recall bias. It is possible that the self-reports may overestimate PA intensity, and IPAQ is more susceptible to this form of bias than other PA questionnaires. However, other studies have also employed self-reported questionnaire for PA intensity assessment. Data was collected by two trained staff, and the results were identical (data not shown). Hence, recall-bias, if any, was minimal and random; and therefore, it was unlikely to influence study results.

In summary, we demonstrated an association between PA and the prevalence of gout among T2DM and HUA patients. Lower PA was associated with a higher prevalence of gout among T2DM and HUA patients, particularly, those of the male sex and those who were drinkers. PA intensity was associated with the prevalence of gout, and the lowest prevalence was achieved from MI-PA.

## Data Availability

The datasets used and analyzed during the current study are available from the corresponding author on reasonable request.
